# Enhanced Ammonia Capture for Adsorption Heat Pumps Using a Salt-Embedded COF Aerogel Composite

**DOI:** 10.3390/gels10120764

**Published:** 2024-11-24

**Authors:** Hiluf T. Fissaha, Duckjong Kim

**Affiliations:** School of Mechanical and Aerospace Engineering, Gyeongsang National University, Jinju 52828, Republic of Korea; teklehiluf53@gmail.com

**Keywords:** covalent organic framework, ammonia adsorption, NaBr-impregnation, adsorption heat pumps, thermal management

## Abstract

Adsorption heat pumps (AHPs) have garnered significant attention due to their efficient use of low-grade thermal energy, eco-friendly nature, and cost-effectiveness. However, a significant challenge lies in developing adsorbent materials that can achieve a high uptake capacity, rapid adsorption rates, and efficient reversible release of refrigerants, such as ammonia (NH_3_). Herein, we developed and synthesized a novel salt-embedded covalent organic framework (COF) composite material designed for enhanced NH_3_ capture. This material was prepared by encapsulating sodium bromide (NaBr) within a porous and densely functionalized sulfonic acid-based COF. The COF was synthesized through a Schiff base (imine) condensation reaction, providing a robust platform for effective NaBr impregnation. The COF-based aerogel composite powder was investigated for its potential in ammonia-based AHPs, benefiting from both the porous, highly functionalized COF structure and the strong NH_3_ affinity of the impregnated NaBr. The composite adsorbent demonstrates an impressive NH_3_ adsorption capacity, adsorption rate, and stability. The exceptional NH_3_ adsorption performance of the COF-based aerogel composite powder is primarily attributed to the uniformly dispersed NaBr within the COF, the coordination of NH_3_ molecules with Na^+^ ions, and the hydrogen bonding interaction between NH_3_ and Br- ions. These findings highlight the potential of the salt-embedded COF composite for use in NH_3_-based AHPs, gas separation, and other related applications.

## 1. Introduction

Adsorption heat pumps (AHPs) utilizing refrigerants as the working fluid offer an environmentally friendly alternative to conventional vapor-compression systems. Ammonia (NH_3_) is highly suitable as a heat pump fluid for several key reasons, such as high latent heat of vaporization, excellent thermodynamic properties, natural and environmentally friendly, self-alarming in case of leakage and good compatibility with metal components like steel and aluminum, which are often used in industrial heat pump systems [1]. However, the performance of AHPs critically depends on the adsorption capacity, kinetics, and thermal stability of the adsorbent material. Metal halides (MHs) have been widely explored as carriers for NH_3_ due to their high NH_3_ uptake capacity and the relatively low energy requirements for NH_3_ release. However, the significant volume changes during sorption–desorption cycles present a challenge in structuring MHs for practical ammonia storage applications [2]. Traditional adsorbents, such as zeolites, silica gels, activated carbon (AC), expanded natural graphite (ENG), carbon nanotubes (CNT), graphene aerogels, and new emerging functional materials such as metal–organic frameworks (MOFs) exhibit limitations in adsorption capacity and long-term thermal stability, necessitating the exploration of advanced materials [3,4,5]. To address this issue, extensive research has been undertaken to develop composite materials that combine metal halides (MHs) with physical adsorbents such as silica, activated carbon (AC), expanded natural graphite (ENG), carbon nanotubes (CNT), and metal-organic frameworks (MOFs) [6,7]. Recently, Shen and Shen [8] developed a copper chloride-impregnated graphite composite (CuCl_2_-G (20)) for NH_3_ sorption. However, the material exhibited relatively low adsorption capacity, and the desorption of ammonia required a high temperature of 200 °C. Joshi and Kim [9] also developed a NaBr impregnated graphene aerogel for ammonia-based adsorption heat pump application. Graphene aerogels face challenges in tailoring their molecular structure and maintaining structural integrity under high NaBr loading. This limitation arises from the lack of functional groups, reduced surface areas, and irregular pore sizes that result from the stacking and aggregation of graphene sheets. Most MOFs, such as ZIF-8 (Zn), exhibit an enhancement in adsorption capacity of approximately 43% when combined with CaCl_2_ [10] while maintaining a high cyclic stability of 99.5%. Nonetheless, the measured adsorption capabilities of MOFs and their composites are suboptimal, and the regeneration challenge restricts their employment in AHPs. Moreover, composites based on ENG-MHs and AC-MHs enhance structural stability; nevertheless, high adsorption rates are constrained by inadequate heat and mass transfer [11].

Therefore, developing efficient and effective adsorbent materials for ammonia-based AHPs (AAHPs) is essential. The primary technical difficulty related to MH-based composite materials is the enhancement of mass transfer and thermal response to improve adsorption rates. Additional research is required to improve the performance of current materials or to create new materials with superior adsorption characteristics, stability, and regeneration conditions to boost the efficacy of AAHPs. Highly porous adsorbents, such as covalent organic frameworks (COFs), have been developed as effective additions to improve the ammonia adsorption capabilities of MHs. COFs have gained considerable attention recently due to their highly ordered porous structures, tunable chemical functionalities, and remarkable stability. These features make COFs promising materials for diverse applications, ranging from gas storage to catalysis and energy storage [12]. However, the traditional synthetic methods for COFs usually demand vacuum conditions, high boiling point organic solvents such as mesitylene or 1,4-dioxane, and long reaction times (48–72 h) [13,14]. More importantly, the resulting COFs are usually formed as powders, which are hardly processable as they are insoluble and infusible. Recently, porous COF aerogels with macropores and inherent micropores have been synthesized by employing scandium triflate (Sc(OTf)_3_) [15] and acetic acid [16]. These COF aerogels and their composites are promising materials for various applications, such as molecular separations [17,18,19], gas adsorption [20], and energy storage [17]. To our knowledge, none of these COF aerogels have been applied to adsorption heat pumps (AHPs). Thus, the application of COF-based aerogels in adsorption heat pumps (AHPs) has sparked interest, particularly for their potential to enhance energy efficiency in sustainable cooling and heating systems.

In this context, we report the development of a NaBr-impregnated sulfonic acid-based COF (SACOF) aerogel powder with enhanced ammonia adsorption capabilities. The impregnation of NaBr within the porous SACOF framework containing sulfur, nitrogen, and oxygen functional group improves the ammonia uptake of the aerogel by forming a strong interaction between the SACOF aerogel and NaBr. Additionally, the flexibility and strong surface forces of the SACOF can help to address issues of swelling and agglomeration of MHs that occur during ammonia adsorption. The lightweight, highly porous structure of the SACOF aerogel powder, combined with the hygroscopic properties of NaBr, provides an ideal platform for optimizing adsorption-driven heat pump technologies. This study presents the synthesis and characterization of the NaBr-impregnated SACOF aerogel, followed by an in-depth evaluation of its performance in ammonia-based adsorption heat pumps. The results demonstrate the material’s potential to overcome the limitations of traditional adsorbents, offering a promising route for advancing energy-efficient thermal management systems.

## 2. Results and Discussion

### 2.1. Design and Preparation of NaBr@SACOF Composite Adsorbent

**Scheme 1:**
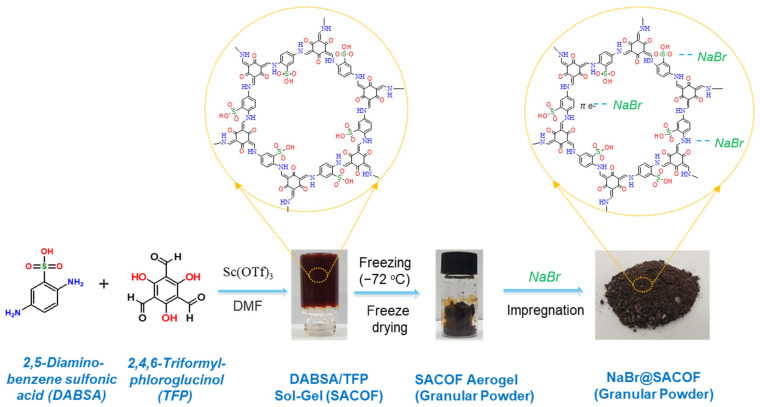
Synthesis scheme of SACOF and NaBr@SACOF.

The SACOF aerogel powder was designed to have secondary amine (R-(NH)-R’), carbonyl (R_2_C=O), and sulfonic acid (R-SO_3_H) functional groups that are the active site for both NaBr and NH_3_ (Scheme 1). This SACOF adsorbent was synthesized via a Schiff base (imine) condensation reaction (Scheme 1). This reaction typically forms β-ketoenamine linkages, which are more stable than simple imines due to keto-enol tautomerism, thus enhancing the COF’s overall stability. The sulfonic acid group (-SO_3_H) on 2,5-Diaminobenzenesulfonic acid does not interfere with the formation of the β-ketoenamine linkages.

Owing to the high catalytic efficiency of scandium triflate (Sc(OTf)_3_) in the aldimine condensation, a dense distribution of COF nuclei was generated rapidly under mild conditions, leading to the formation of the SACOF gel within 10 min. This demonstrates effective control of COF morphology based on the nucleation rate. The rapid reaction kinetics and gel formation, superior to those catalyzed by acetic acid [15], highlight the efficiency of Sc(OTf)_3_ at room temperature. A specific concentration of NaBr was infiltrated into the porous structure of the SACOF framework, resulting in NaBr@SACOF-30 at 1 M NaBr, NaBr@SACOF-70 at 3 M NaBr, and NaBr@SACOF-80 at 5 M NaBr. Following freeze-drying, NaBr recrystallized and was uniformly distributed throughout the pores of the SACOF aerogel (Scheme 1). The successful synthesis of the SACOF aerogel powder and NaBr@SACOF-80 composite adsorbent was verified using standard chemical and spectroscopic characterization techniques, as presented in Section 2.2.

### 2.2. Characterization of SACOF Aerogel Powder and NaBr@SACOF-80 Composite Adsorbent

**Figure 1:**
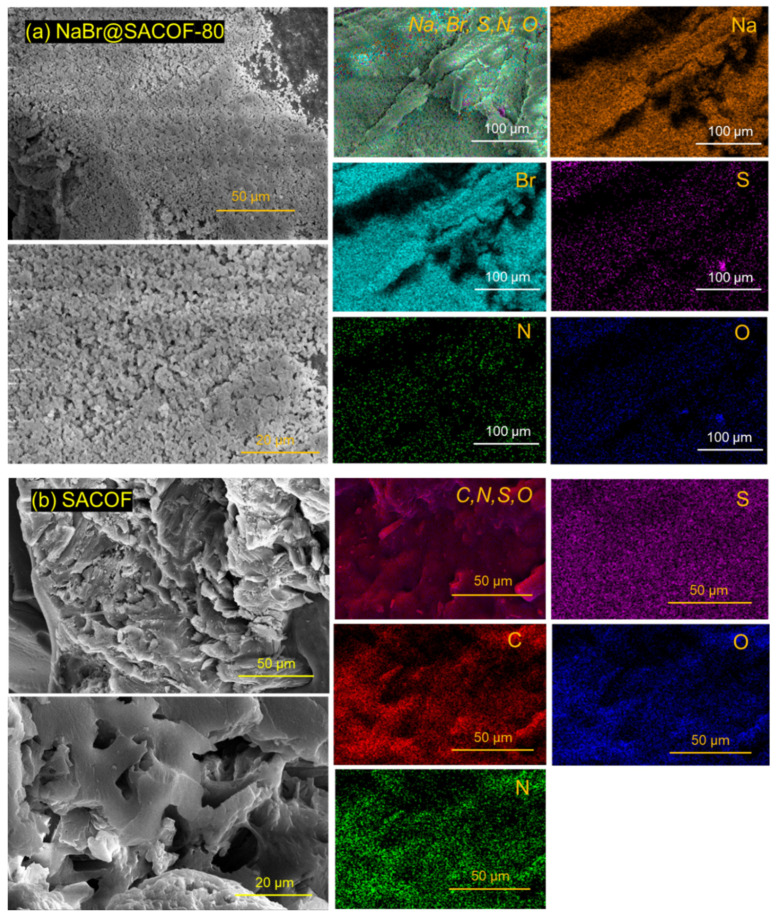
Field emission scanning electron microscopy (FE-SEM) analysis of (**a**) NaBr@SACOF-80 and (**b**) SACOF at both low and high magnification, along with corresponding energy dispersive spectroscopy (EDS) analysis of selected elements.

The FESEM images at different magnifications (Figure 1) clearly depict well-dispersed NaBr crystals on the surface of the SACOF networks. SEM analysis (Figure 1a) revealed that the NaBr@SACOF-80 composite material exhibits a granular texture consisting of cylindrical-like meso- and macropores ranging from 0.8 to 1.9 μm. The SACOF (Figure 1b) revealed a porous, rough, and uneven surface with a complex structure featuring various ridges, valleys, and protrusions. These images also showed a heterogeneous distribution of pores, with sizes ranging from sub-micron to larger voids (1.3 to 7.3 μm), indicating a hierarchical pore structure. This interconnected pore network provides potential pathways for NaBr impregnation and NH_3_ adsorption while enhancing properties such as mechanical strength and surface area. Elemental mapping also highlighted the distribution of C, N, S, and O across the SACOF matrix, further emphasizing the material’s multifunctional characteristics. Compared to the pristine SACOF (Figure 1b), the visible pores in the composite are fully covered by NaBr. The distribution of small NaBr crystallites on the high-surface-area SACOF network significantly increases the available sites for ammonia−NaBr interactions during adsorption, resulting in an enhanced adsorption capacity. The presence of Na and Br atoms in the SACOF surface was further confirmed via EDAX mapping (Figure 1a), which showed that NaBr was highly dispersed throughout the SACOF matrix. Elemental distribution mapping with EDX confirmed the presence of Na and Br from NaBr and S, N, and O from the SACOF. The Na and Br ions were found to interact with the SACOF functional groups, including aromatic π electrons and lone pair electrons from N, S, and O.

**Figure 2:**
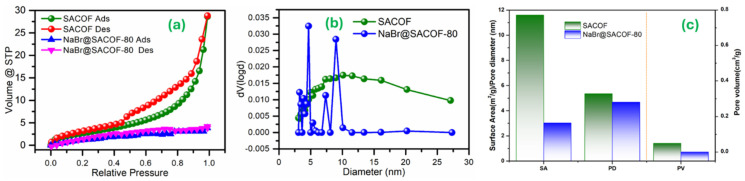
BET analysis of NaBr@SACOF-80 and SACOF. (**a**) N_2_ adsorption isotherms, (**b**) pore size distribution, and (**c**) surface area, pore size, and pore volume.

N_2_ adsorption/desorption curves (Figure 2a) of SACOF exhibit typical type IV isotherms with clear mesoporosity and a hysteresis loop at p/po~0.4–0.9, indicating its mesoporosity [21]. While NaBr@SACOF-80 shows limited adsorption, indicating a structural modification after the introduction of NaBr (Figure 2a). Pore size distributions (Figure 2b) reveal peak pore diameters of ∅p~10.0 nm for SACOF and ∅p~5.0 nm for NaBr@SACOF-80 from BJH. SACOF has a significantly higher surface area (~11.6 m^2^/g), while NaBr@SACOF-80 shows a marked reduction (~3.0 m^2^/g). This suggests that incorporating NaBr reduces the overall surface area of SACOF. The pore diameter of SACOF is also larger than that of NaBr@SACOF-80. However, the difference is less drastic than in the surface area (Figure 2c). SACOF has a pore diameter of approximately 5.4 nm, while NaBr@SACOF-80 has a slightly lower pore diameter of around 4.7 nm. Both materials exhibit small pore volumes, but SACOF again shows a higher value (~0.05 cm^3^/g), while NaBr@SACOF-80 has a lower pore volume (~0.001 cm^3^/g). The incorporation of NaBr into SACOF results in a reduction of surface area, pore diameter, and pore volume. This likely indicates that the NaBr is occupying or filling the pores of SACOF, thereby reducing the available space for adsorption. This can be a trade-off where NaBr enhances certain properties (e.g., adsorption capacity for specific applications) at the expense of surface area and porosity.

SEM and BET data cumulatively suggest that the porous structure of the NaBr@SACOF-80 composite played a crucial role in enhancing its adsorption capacity and adsorption/desorption rates compared to the pure NaBr structure.

**Figure 3:**
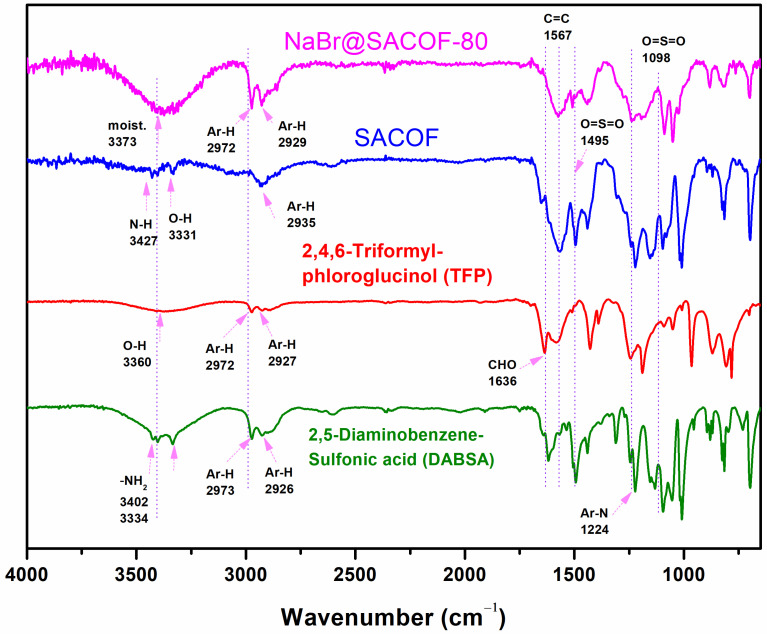
FTIR analysis of NaBr@SCCOF-80 and its intermediates.

The FTIR spectra of the pristine SACOF and the composite adsorbent (NaBr@SACOF-80) give a characteristic peak of the imine bond appearing at around 1620 cm^−1^ (Figure 3). NaBr@SACOF shows additional peaks (1567 cm^−1^, 1495 cm^−1^, 1098 cm^−1^) associated with unsaturated carbon bonds (C=C) and sulfonate (O=S=O) compared to SACOF, indicating the successful incorporation of NaBr and sulfonate functionalities. SACOF and NaBr@SACOF-80 share many common peaks, particularly in the aromatic C–H region (2935 cm^−1^) and the sulfonate group region, confirming that SACOF is the base material in the NaBr@SACOF-80 composite. TFP shows distinctive peaks for hydroxyl (3360 cm^−1^) and aldehyde (1636 cm^−1^) groups, suggesting a different chemical structure compared to SACOF and NaBr@SACOF-80. DABSA has strong amine (–NH_2_) peaks (3402 cm^−1^, 3334 cm^−1^) and aromatic nitrogen (1224 cm^−1^) peaks, distinguishing it from the other samples. Therefore, FTIR analysis confirmed the successful synthesis of the SACOF aerogel powder and the NaBr@SACOF-80 composite adsorbent. 

**Figure 4:**
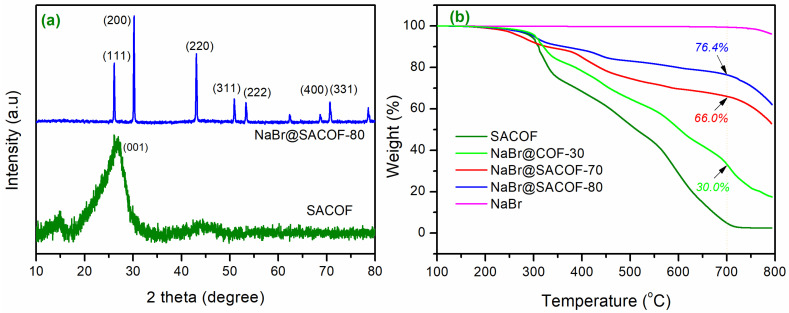
Structural and thermal analysis of NaBr@SACOF-80 and its intermediates (**a**) XRD and (**b**) TGA analysis.

The X-ray diffraction (XRD) pattern of SACOF exhibits a broad peak at approximately 20° 2θ, corresponding to the (001) plane, indicating that SACOF possesses an amorphous or poorly crystalline structure (Figure 4a). In contrast, the diffraction pattern of NaBr@SACOF displays sharp peaks, which are indicative of a crystalline structure, with distinct planes such as (111), (200), (220), (311), (222), (400), and (331). These planes correspond to the crystal planes of NaBr [9], confirming the successful incorporation of NaBr into SACOF (Figure 4a). Therefore, SACOF is predominantly amorphous, whereas NaBr@SACOF-80 exhibits a crystalline structure characteristic of NaBr.

Thermogravimetric analysis (TGA) of SACOF reveals significant weight loss, with a final residue of 2.8% at approximately 700 °C (Figure 4b). This substantial weight loss suggests the decomposition or volatilization of organic material. In contrast, NaBr@SACOF-30, NaBr@SACOF-70, and NaBr@SACOF-80 exhibit significantly lower overall weight loss than pure SACOF. The final calculated residues (accounting for both the SACOF residual and the weight loss of NaBr at ~700 °C) are 30.1%, 69.6%, and 80.1%, respectively. This indicates the presence of NaBr, which remains thermally stable at these temperatures. NaBr alone exhibits minimal weight loss, retaining 94.2% of its weight, further demonstrating its thermal stability up to 700 °C. The TGA curves suggest that SACOF undergoes extensive thermal degradation, while NaBr@SACOFs demonstrate enhanced thermal stability due to the presence of NaBr (Figure 4b). Notably, the thermal stability of the composite increases with higher NaBr content. Therefore, XRD and TGA analysis confirmed the successful preparation of the NaBr@SACOF composite adsorbent.

### 2.3. NH_3_ Adsorption/Desorption Properties of NaBr@SACOF

The NH_3_ adsorption experiments were conducted on an empty reactor chamber as well as on various samples, including SACOF, pure NaBr, and NaBr@SACOF composites with NaBr loadings of 30%, 70%, and 80%, according to the experimental conditions outlined in Section 4.5. All samples exhibited the ability to fully desorb ammonia at 80 °C, partially adsorb ammonia at 40 °C, and achieve complete adsorption at 20 °C. Consequently, the NH_3_ adsorption experiments for all samples were carried out at these specific temperatures.. The mass of the adsorbed NH_3_ gas was calculated from the pressure and temperature data collected during the adsorption run (Figure S1) using the ideal gas equation (Equation (1)).

(1) $$m_{{NH}_{3}}\left(g\right)=\frac{V_{r}}{R}\times \left(\frac{P_{r}\left(0\right)}{T_{1}\left(0\right)}-\frac{P_{r}\left(t\right)}{T_{1}\left(t\right)}\right)$$

where *R* represents the NH_3_ gas constant (488.21 J/kg·K), *P_r_*(0) denotes the experimentally recorded initial pressure, and *T*_1_(0) indicates the initial temperature. *P*_r_(*t*) represents the pressure in the reactor at specific time *t*, and *T_1_*(*t*) is the temperature at that corresponding time *t* in seconds. The adsorption capacity of the samples was also calculated as the ratio of the mass of adsorbed NH_3_ (mNH_3_) to the mass of the dry adsorbent (m-adsorbent, g) Equation (2).

(2) $$Adsorption\;capacity\left(q;\frac{g}{g}\right)=\frac{m_{{NH}_{3}}}{m_{NaBr@SACOF}}$$

**Figure 5:**
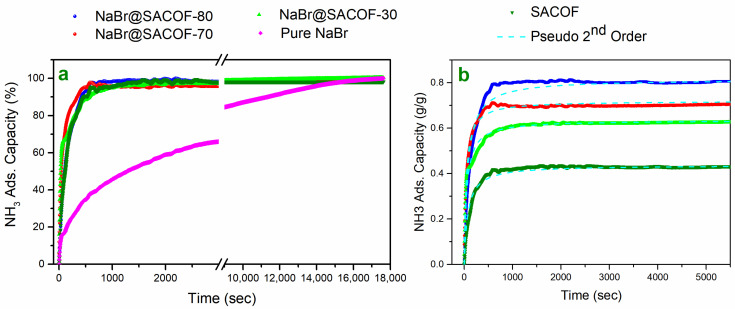
Adsorption characteristics of NaBr@SACOF composites, SACOF, and pure NaBr: (**a**) amount of adsorbed NH_3_ (%) between the temperature range of 80 °C to 20 °C; (**b**) time profile for NaBr@SACOF-80, SACOF, and pure NaBr; and the fitted non-linear kinetic model (N.B: better fitted model only shown here).

The adsorption kinetics of NH_3_ for various materials over time are illustrated in Figure 5a. Within the temperature range of 80 °C to 20 °C, the initial adsorption rate is notably high for all materials except pure NaBr, indicating rapid NH_3_ uptake by most samples. Composite materials, such as NaBr@SACOF-80, NaBr@SACOF-70, and NaBr@SACOF-30, exhibit significantly faster adsorption rates compared to pure NaBr. Furthermore, all NaBr@SACOF composites and SACOF reach near saturation (~100% adsorption) in a relatively short time, demonstrating their high adsorption efficiency. In contrast, pure NaBr approaches equilibrium only after a prolonged period, highlighting its slower adsorption kinetics (Figure 5a). A similar trend is evident in the temperature range of 80 °C to 40 °C, as shown in Figure S2. In this case, NaBr@SACOF-80, NaBr@SACOF-70, and NaBr@SACOF-30 achieve higher adsorption capacities more rapidly than SACOF and pure NaBr, further underscoring the superior performance of NaBr@SACOF composite adsorbents.

To better understand the underlying uptake rate mechanism, results were fitted through different kinetic models. The pseudo-second order kinetic models expressed in Equation (3) provides better fitting among others, where *k*_2_ (g g^−1^ s^−1^) is the rate constant of the model [22].

(3) $$q_{t}=\frac{k_{2}q_{e}^{2}t}{1+k_{2}q_{e}t}$$

Regression analysis (Table 1) demonstrates that the uptake rate of NH_3_ can be effectively described by the pseudo-second-order kinetic model. In the initial phase of adsorption, all adsorbents exhibited a sharp increase in NH_3_ adsorption capacity within the first 500 s, suggesting rapid adsorption likely due to the abundant availability of active sites (Figure 5b). Beyond approximately 1000 s, the adsorption curves plateau, indicating the attainment of equilibrium. Almost all samples reached equilibrium within approximately 20 min, a finding consistent with the predictions of the pseudo-second-order model (Table 1). Moreover, the equilibrium adsorption capacities (*q_e_*) derived from the model closely aligns with the experimental data. These results affirm that the pseudo-second-order model provides an accurate representation of the adsorption kinetics for all samples (Figure 5b), implying that the adsorption processes are predominantly driven by chemisorption.

The pseudo-second-order model offers valuable insights into the relationship between the adsorption rate constant (*k_2_*) and the equilibrium adsorption capacity (Table 1). Materials with higher adsorption capacities, such as NaBr@SACOF-80, tend to exhibit lower *k*_2_ values, suggesting slower adsorption kinetics but greater overall capacity. Conversely, materials with faster adsorption rates, such as NaBr@SACOF-70, reach equilibrium more quickly but display slightly reduced capacities, possibly due to fewer adsorption sites or lower NH_3_ affinity. The functionalization with NaBr significantly influences both adsorption rates and capacities. NaBr likely enhances the chemical interactions between NH_3_ molecules and the adsorbent, resulting in increased capacities, as observed in NaBr@SACOF-80. However, excessive NaBr functionalization may reduce *k_2_* due to potential steric hindrance or slower diffusion of NH_3_ to active sites. Since the adsorption rate of NaBr@SACOF-80 is not significantly lower than the other composite materials, it is the optimal choice for adsorption heat pump application that requires both a high adsorption capacity and rapid adsorption rate. Pristine SACOF demonstrates relatively fast adsorption kinetics, with second-order rate constant (*k_2_*) values comparable to those of NaBr@SACOF-30 and notably higher than those of pure NaBr (Figure S3). However, it exhibits a significantly lower adsorption capacity, highlighting the essential role of NaBr functionalization in enhancing both the adsorption performance and overall effectiveness of the material.

**Figure 6:**
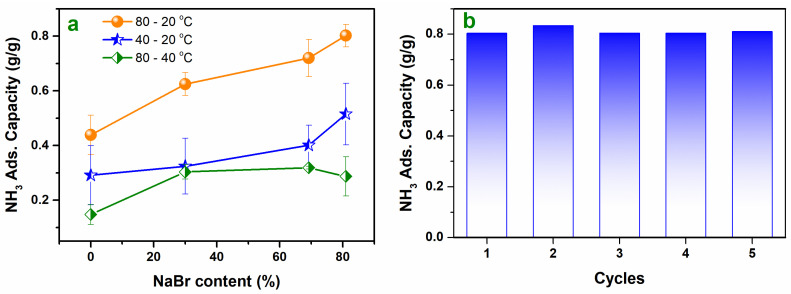
Adsorption characteristics: (**a**) adsorption capacity of SACOF as a function of NaBr weight percentage and adsorption temperature and (**b**) cyclic NH_3_ adsorption capacity of NaBr@SACOF-80.

The variation in adsorption behavior is further evident when considering the adsorption capacities of these materials across specific temperature ranges, such as 40–20 °C. The adsorption capacity of each adsorbent within the 40–20 °C temperature range (Figure 6a) was determined by subtracting the adsorption capacity measured in the 80–40 °C range from that measured in the 80–20 °C range. This temperature (40–20 °C) range is critical for low-temperature thermal energy applications, including refrigeration, heat pumps, and energy storage. These systems often operate near ambient temperatures, where efficient low temperature desorption of working fluids, such as NH_3_, directly enhances energy conversion efficiency. Effective desorption at low temperature facilitates the utilization of low-grade thermal energy, which is abundant yet underutilized due to its limited potential for direct energy conversion. In the 40–20 °C range, the adsorption capacity of each adsorbent increases with NaBr content, following the trend: SACOF (0.29 g/g) < NaBr@SACOF-30 (0.32 g/g) < NaBr@SACOF-70 (0.40 g/g) < NaBr@SACOF-80 (0.52 g/g). This demonstrates that the material’s sorption properties benefit from NaBr addition at lower temperature gradients, though less dramatically than at higher gradients. Thus, NaBr@SACOF-80 demonstrates superior performance due to its optimized adsorption properties combined with the highest NaBr content (80.1%). Across the 40–20 °C range, this material achieves the highest NH_3_ adsorption capacity, maximizing its potential for thermal energy utilization. Its enhanced performance in the critical 40–20 °C range makes it particularly suitable for low-temperature applications, such as waste heat recovery and solar thermal energy utilization, where efficient energy management is essential.

The adsorption of NH_3_ on NaBr@SACOF-80 is likely governed by specific interactions between NH_3_ molecules and the active sites provided by the composite material. Sodium ions (Na⁺) in NaBr serve as Lewis acid sites, which can interact with the lone pair of electrons on the nitrogen atom of NH_3_, forming a coordinate bond [23]. This bond enhances the affinity of NH_3_ for the composite surface. Bromide ions (Br^−^), being highly polarizable, may also contribute to dipole-induced dipole interactions with the NH_3_ molecule. Sulfonic acid groups (-SO_3_H) in the SACOF framework provide hydrogen bonding sites. NH_3_, acting as a hydrogen bond acceptor, can interact with these groups through the lone pair on its nitrogen atom. The porous structure of SACOF offers a high surface area, facilitating physical adsorption via van der Waals forces. These forces are non-specific but play a significant role in capturing NH_3_ molecules within the pores. The presence of NaBr likely enhances the adsorption performance due to the combined effects of ionic (Na⁺ and Br^−^) and sulfonic acid sites. These create a dual-functional material capable of adsorbing NH_3_ through multiple mechanisms, increasing the adsorption capacity.

The cyclic stability of the NaBr@SACOF-80 structure was evaluated over five cycles by subjecting it to repeated high-temperature transitions, from complete adsorption at 20 °C to complete desorption at 80 °C. Ammonia desorption occurs primarily through the application of thermal energy. When the material is heated, typically to around 80 °C, the thermal energy overcomes the adsorption forces (e.g., physisorption or weak chemisorption) binding NH_3_ to the material. This allows the NH_3_ molecules to desorb and leave the structure, ensuring the material’s regeneration for subsequent adsorption–desorption cycles. The process is designed to be reversible to maintain cyclic stability. The reactor gauge pressure, initially approximately 8.5 bar at 80 °C, dropped to 5.5 bar at 20 °C due to adsorption by the sample, and then returned to 8.5 bar with the temperature increase to 80 °C. NaBr@SACOF-80 exhibited exceptional cyclic stability (~99.9%) in terms of NH_3_ adsorption/desorption capacity, adsorption/desorption rate, and structural integrity. The NH_3_ sorption capacity in the first cycle was recorded at ~0.80 g/g, which increased to ~0.83 g/g in the second cycle and maintained a consistent range of 0.80 to 0.81 g/g in subsequent cycles (Figure 6b). This slight increase in adsorption capacity is attributed to the recrystallization and size reduction of the impregnated NaBr, which provides additional surface sites for ammonia adsorption in later cycles. NaBr@SACOF-80 demonstrated excellent cyclic stability, underscoring its potential as an effective adsorbent for NH_3_ capture.

**Figure 7:**
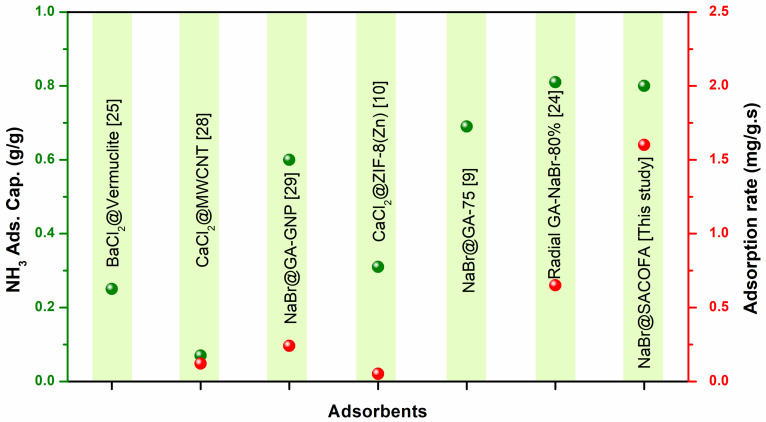
Adsorption properties of the as-prepared NaBr@SACOF compared with the literature [9,10,24,25,28,29].

A comprehensive comparison of the adsorption properties of the synthesized NaBr@SACOF-80 composite with those reported in the literature was conducted (Figure 7). Radial GA-NaBr 80% exhibited the highest adsorption capacity, along with a moderate adsorption rate [24]. However, the interaction between NaBr and graphene aerogel is primarily physical, and the NaBr crystals do not form strong bonds with the graphene aerogel. Vermiculite@BaCl_2_, as reported by Veselovskaya et al. (2010) [25], demonstrated a high adsorption rate but a relatively lower adsorption capacity. Metal–organic frameworks (MOFs), such as CaCl_2_@ZIF-8(Zn), have shown promise due to their adsorption properties; however, they lack some of the critical attributes required for adsorption heat pump (AHP) applications [10,26,27]. Although MOF-based adsorbents boast a high adsorption capacity, they suffer from poor cyclic stability and a low sorption rate, with the additional challenge of difficult MOF regeneration [26].

Similarly, CaCl_2_@ZIF-8(Zn) has demonstrated good adsorption capacity but is hindered by limited cyclic stability and an insufficient sorption rate [10]. In contrast, the NaBr@SACOF-80 composite, designed specifically for applications in ammonia-based adsorption heat pumps (AAHPs), demonstrated exceptional adsorption performance under high-temperature differentials (80 °C to 20 °C). This composite achieved a high sorption capacity of 0.80 g/g and an impressive adsorption rate of 1.60 mg/g/s at 90% of its adsorption capacity. These findings suggest that the NaBr@SACOF composite holds significant potential as a highly effective low-grade heat adsorbent for AAHP applications, surpassing previously reported materials. The adsorption properties, namely, adsorption capacity, adsorption rate, and cyclic stability, significantly influence thermal energy management systems, such as adsorption heat pumps and thermal energy storage [24]. Improving adsorption capacity enhances heat transfer capabilities within adsorption heat pumps and increases energy density in thermal energy storage systems. Likewise, a higher adsorption rate boosts the specific cooling and heating power of the heat pump while enabling faster thermal energy charging and discharging in thermal energy storage. Furthermore, enhanced cyclic stability of the adsorbent ensures sustained long-term performance in heat pumps and extends the operational lifespan and efficiency of thermal energy storage systems.

## 3. Conclusions

This study discusses the design and development of a composite material, a NaBr-impregnated highly porous SACOF, for potential application in AAHPs. These porous NaBr@SACOF composites offer remarkable properties, such as a wide range of NaBr loading up to 80 wt%. They also exhibited enhanced adsorption characteristics for NaBr by facilitating mass transfer and mitigating swelling and agglomeration concerns. The NaBr@SACOF-80 composite demonstrated a high adsorption capacity and rapid adsorption and desorption rates due to its elevated surface area, porosity, and affinity for ammonia. Moreover, the NaBr@SACOF-80 composite provides remarkable corrosion resistance and adaptability in constructing adsorbent beds for AHP applications. Consequently, due to its superior structural stability and remarkable adsorption characteristics, the NaBr@SACOF-80 composite possesses the potential to serve as an efficient adsorption medium for high-performance ammonia-based adsorption heat pumps.

## 4. Materials and Methods

### 4.1. Materials and Reagents

2,5-Diaminobenzenesulfonic acid (≥97.0%, DABSA), N,N-Dimethylformamide (≥99.8%, DMF), ethanol (99.5%, EtOH), and scandium(III)triflate (99%, Sc(OTf)3) were purchased from Sigma-Aldrich (St. Louis, MO, USA). 2,4,6-Triformylphloroglucinol (>98.0% TFP) was purchased from Thermo Fisher Scientific (Seoul, Korea). Acetone (99.5%, AcOH) was manufactured by Samchun Chemicals Co., Ltd. (Pyeongtaek-Si, Korea). Deionized (DI) water (18.2 mΩ cm^−1^ at 25 °C) was processed through a Millipore Milli-Q system. All the solvents and reagents in this study were used without further purification.

### 4.2. Synthesis of DABSA/TFP COF(SACOF)

SACOF aerogel powder was synthesized via a Schiff base (imine) condensation reaction (Scheme 1). Specifically, 2,4,6-Triformylphloroglucinol and 2,5-Diaminobenzenesulfonic acid were added to a 25 mL Pyrex beaker containing dimethylformamide (DMF). Subsequently, scandium triflate (Sc(OTf)_3_) catalyst was added, and the mixture was sonicated until a homogeneous solution was achieved. The mixture was left undisturbed, and the gel formation could be observed after several minutes. After a reaction for 24 h, the formed gel was treated with DMF, THF, acetone, ethanol, and water successively and then freeze-dried for two days to obtain pure SACOF aerogel coarse powder.

### 4.3. Preparation of NaBr@SACOF

The SACOF sample was vacuum-dried at 110 °C for 8 h and then cooled to ambient temperature. To prepare the NaBr@SACOF composite adsorbent, a specified amount of NaBr crystals was first dissolved in deionized water. Subsequently, a measured amount of the dried SACOF sample was immersed in 25 mL of NaBr solution at varying concentrations (1 M, 3 M, and 5 M). The impregnation was conducted in a vacuum oven at 40 °C for 6 h. Following impregnation, the NaBr-loaded sample was separated from the solution via decantation and then frozen. The frozen sample was subsequently freeze-dried for two days and further dried in a vacuum oven at 110 °C for an additional 6 h. Based on the amount of NaBr impregnated in the composite (by weight percentage), the resulting composites were designated as NaBr@SACOF-30, NaBr@SACOF-70, and NaBr@SACOF-80.

### 4.4. Characterization

Nitrogen adsorption and desorption isotherms were measured at 77.5 K using an ASiQwin Quantachrome instrument. The samples were treated at 110 °C for 8 h before measurements. The surface area was measured via the Brunauer–Emmett–Teller (BET) model, while the pore size distribution was processed through the Barrett–Joyner–Halenda (BJH) models. Thermogravimetric analysis was carried out on a TGA TA instrument (SDT 650) in the air at a 10 °C min^−1^ heating rate to determine the NaBr loading content of NaBr@SACOF. Power X-ray diffraction (PXRD) data were obtained with a multipurpose X-ray diffractometer (Bruker (D8 Advance A25 Plus)) at 40 kV and 40 mA with Cu Kradiation from 2θ = 10° to 80° in 0.05° increments. Fourier transform infrared spectra (FTIR) of the samples were collected on a spectrum 400 spectrometer (Thermo Fisher (IS50)). Structural and morphological properties of the samples were examined using a Thermo (Apreo S) scanning electron microscope (SEM).

### 4.5. NH_3_ Adsorption Experiments

**Figure 8:**
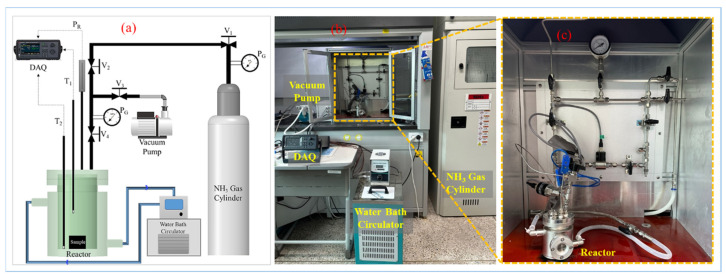
(**a**) Schematic layout of our customized high-pressure ammonia sorption analyzer, (**b**) actual picture of the experimental setup, and (**c**) high-pressure ammonia reactor with pressure and temperature sensors [29].

The NH_3_ adsorption setup used in this study consists of an NH_3_ cylinder, a reactor chamber, a vacuum pump, a water bath circulator, valves (*V*_1_–*V*_4_), temperature sensors (*T*_1_ and *T*_2_), a pressure sensor (*P_r_*), and a data acquisition system (DAQ) connected to a computer (Figure 8). The reactor is a stainless-steel chamber with an internal diameter of 45 mm and a depth of 70 mm, yielding a total volume (*V_r_*) of 125 mL. It is connected to an NH_3_ gas cylinder with appropriate valves and a vacuum pump. Calibrated thermocouples are used to monitor temperatures within the reactor chamber—one thermocouple (*T*_1_) is positioned at the center of the reactor to measure chamber temperature, while another (*T*_2_) is placed at the bottom to record the sorbent temperature. Additionally, a pressure sensor (*P_r_*) monitors the NH_3_ pressure within the reactor (Figure 8).

Before adsorption experiments, all samples were subjected to vacuum drying at 110 °C for 8 h to eliminate moisture and other adsorbed gases. The SACOF and NaBr@SACOF samples were placed at the bottom of the reactor. Subsequently, the reactor was sealed with a solid casing with a thickness of 10 mm on top and 4 mm on the sides. After checking the setup for leaks by creating a vacuum (*V*_3_, *V*_4_—open and *V*_1_, *V*_2_—closed), the entire reactor was heated to 80 ± 0.5 °C using the thermostatic water bath circulator connected to the reactor (Figure 8). The appropriate amount of NH_3_ gas is then charged into the reactor for analysis (*V*_1_, *V*_2_, *V*_4_—open, and *V*_3_—closed) after a temperature of 80 ± 0.5 °C has been reached inside the reactor. The reactor is then cooled via the water bath circulator until it reaches 40 and 20 °C (Figure 8). The experimental data of variations in the pressure (*P_r_*) and temperatures (*T*_1_ and *T*_2_) of the NH_3_ gas inside the reactor were collected using DAQ at regular intervals of 3 s while the reactor was cooled down from 80 ± 0.5 to 40 ± 0.5 °C and 80 ± 0.5 to 20 ± 0.5 °C.

A baseline was also created by measuring adsorption in the empty reactor under conditions identical to the samples. The adsorption capacity of the empty reactor served as a reference for the sample. For the blank measurements, the vacant reactor chamber was heated to an initial temperature of 80 ± 0.5 °C using a water bath equipped with a PID controller. Subsequently, the reactor was subjected to an ammonia gas flow, and the initial pressure was meticulously set at around 7.2 bar (gauge pressure). Upon establishing equilibrium at 80 °C and 7.2 bar pressure, the reactor temperature is promptly decreased to 40 ± 0.5 °C and 20 ± 0.5 °C using a separate water bath pre-set to each temperature. Upon stabilization of the gas pressure in the cooled chamber, the ammonia pressure reduction was quantified using a pressure sensor and recorded via a data-collecting device (Keysight, DAQ970A). Using the principles of the ideal gas law (Equation (1)), the gathered manometric data were subsequently transformed into the appropriate mass of adsorbed ammonia. The actual ammonia adsorption by the samples was ascertained using blank test subtraction. The quantity of adsorbed NH_3_, after deducting the blank test results, was divided by the sample mass, resulting in the adsorption capacity of the examined sample (Equation (2)). The margin of error (MOE) associated with the NH_3_ adsorption experiment was determined using Student’s *t*-test method for two trials, as demonstrated in Equation (4).

(4) $$MOE=\frac{t_{cr(95\%)}\times s}{\sqrt{n}}$$

where *t* represents the Student’s *t*-value for a 95% confidence interval, set at 12.706, which accounts for two trials (degrees of freedom = 1), *s* denotes the standard deviation, and *n* is the sample size (two trials), which represents the variability across the dataset.

## Figures and Tables

**Table 1:** Adsorption kinetic coefficients of SACOF at different NaBr contents.

Samples	Pseudo-Second Order		
	*q_e_* (g g^−1^)	*k*_2_ × 10^−2^ (g g^−1^ s^−1^)	*r^2^*
NaBr@SACOF-80	0.816	1.73	0.94
NaBr@SACOF-70	0.720	3.45	0.93
NaBr@SACOF-30	0.636	3.09	0.95
SACOF	0.437	3.04	0.95

Conditions [Po = 6.2 bar; m ≅ 120 mg; T = 20/80 °C].

## Data Availability

The original contributions presented in this study are included in the article/Supplementary Material. Further inquiries can be directed to the corresponding author.

## Supplementary Materials

The following supporting information can be downloaded at: https://www.mdpi.com/article/10.3390/gels10120764/s1, Figure S1: NH_3_ adsorption pressure (bar) and corresponding sample and gas temperature (°C) profiles over time; Figure S2: Amount of adsorbed NH₃ (%) of NaBr@SACOF composites, SACOF, and pure NaBr between the temperature range of 80 °C to 40 °C; Figure S3: Time profile for pure NaBr and the fitted non-linear kinetic model between the temperature range of 80 °C to 20 °C.
