# Supplementary material for: Enhanced Ammonia Capture for Adsorption Heat Pumps Using a Salt-Embedded COF Aerogel Composite
Source: Gels. 2024 Nov 24;10(12):764. doi: 10.3390/gels10120764 (PMC11675208; doi:10.3390/gels10120764)
Supplement: Supplementary file 1 [file gels-10-00764-s001.zip › gels-3326577-supplementary.pdf]

Supporting Information

Enhanced Ammonia Capture for Adsorption Heat Pumps Using a Salt-Embedded COF Aerogel Composite

Hiluf T. Fissaha and Duckjong Kim*

School of Mechanical and Aerospace Engineering, Gyeongsang National University, Jinju 52828, Republic of Korea.

* Correspondence: dkim@gnu.ac.kr: orcid.org/0000-0001-6372-8785

1. *NH_3 adsorption pressure (bar) and corresponding sample and gas temperature (°C) profiles over time*

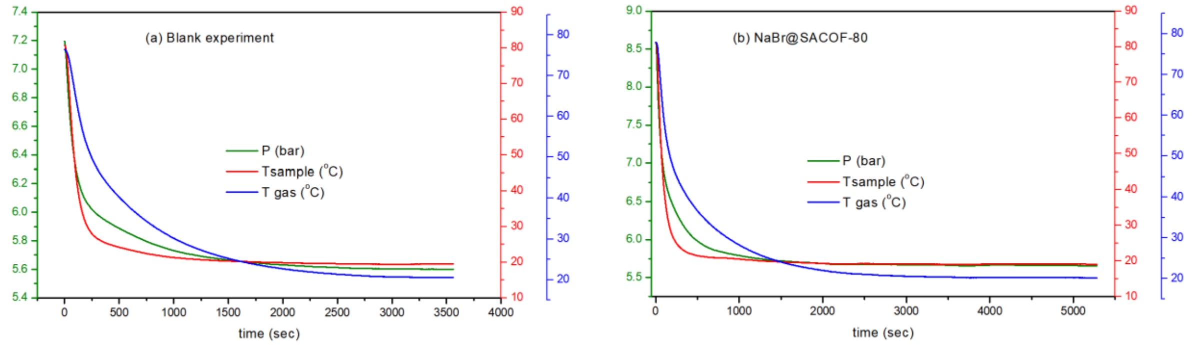

Figure S1. NH_3 adsorption pressure (bar) and corresponding sample and gas temperature (°C) profiles over time for (a) blank experiment, and (b) NaBr@SACOF-80.

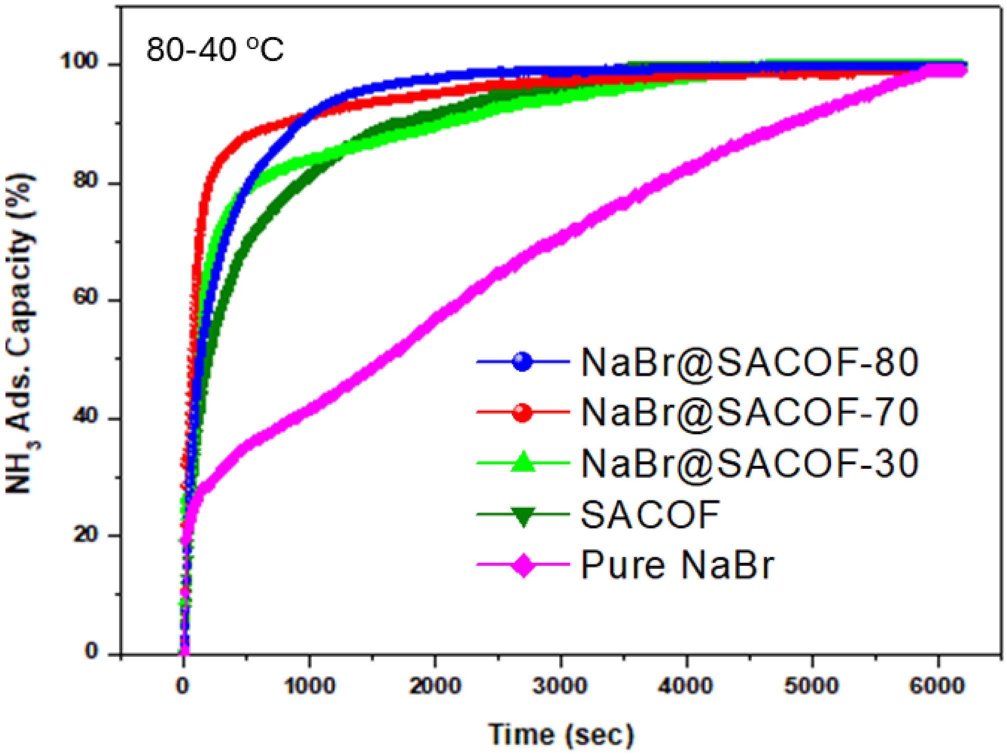

Figure S2. Amount of adsorbed NH_3 (%) of NaBr@SACOF composites, SACOF, and pure NaBr between the temperature range of 80 °C to 40 °C.

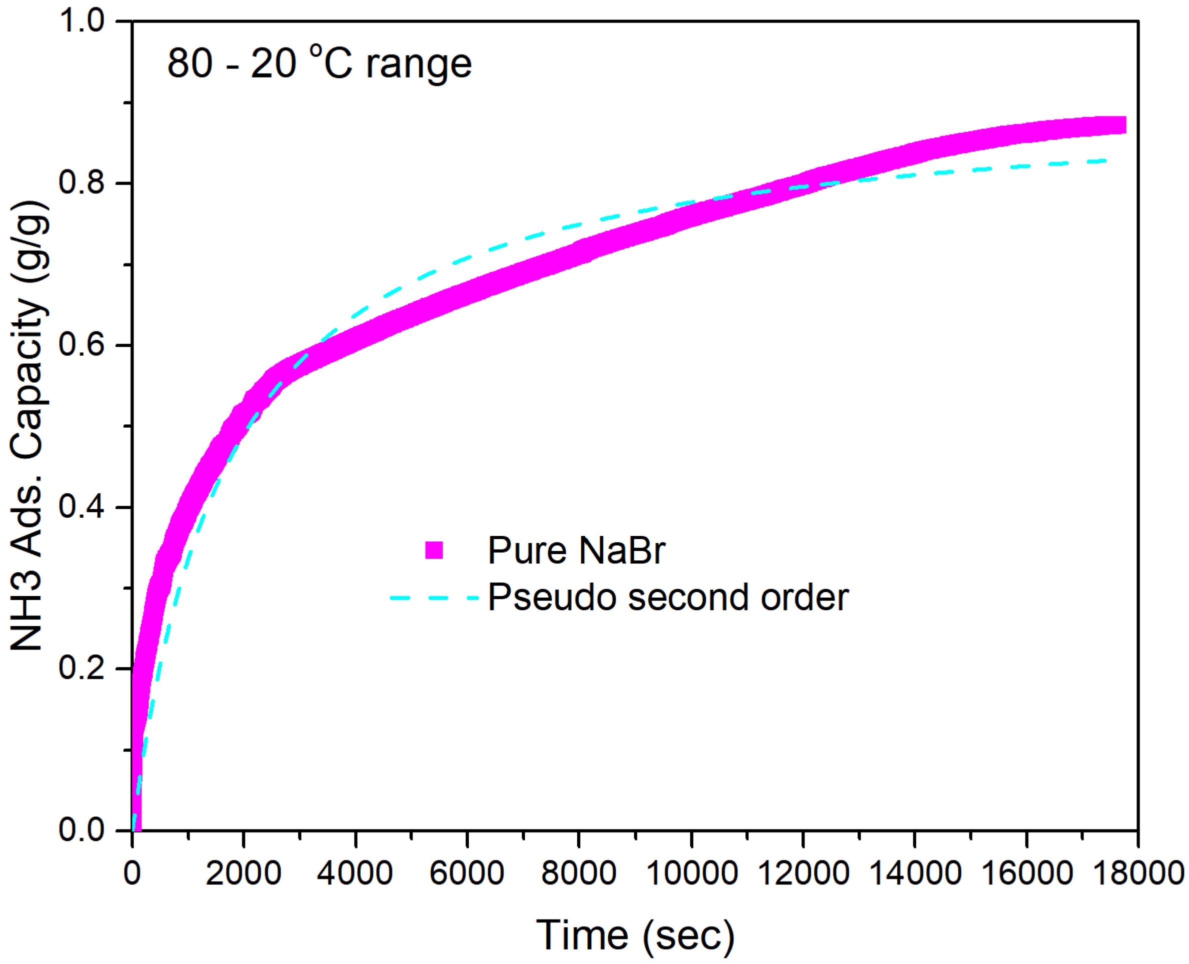

Figure S3. Time profile for pure NaBr and the fitted non-linear kinetic model between the temperature range of 80 °C to 20 °C. (N.B: better fitted model only shown here).
